# Supplementary material for: Construction of a Heterotrophic Nitrification–Aerobic Denitrification Composite Microbial Consortium and Its Bioaugmentation Role in Wastewater Treatment
Source: Biology (Basel). 2025 Dec 4;14(12):1734. doi: 10.3390/biology14121734 (PMC12731035; doi:10.3390/biology14121734)
Supplement: Supplementary file 1 [file biology-14-01734-s001.zip › biology-3977670-supplementary.pdf]

Supplementary Materials

PCR amplification of 16S rRNA gene V3-V4 hypervariable regions in two SBRs across operational phases

Amplification of the V3-V4 hypervariable regions (target size: ~468 bp) from sludge samples was performed using primers 338F and 806R , as validated by agarose gel electrophoresis (Figure. 4). All eight samples collected across four operational phases(A1–D2) exhibited amplicons of the expected size (~468 bp), confirming successful target amplification. Nucleic acid concentrations, quantified via Nanodrop 2000 spectrophotometer(Thermo Fisher Scientific, Waltham, MA, USA), demonstrated sufficient concentration (20–100 ng/μL) and purity (A260/A280 ratio 1.8–2.0) to meet Illumina library construction and sequencing requirements.

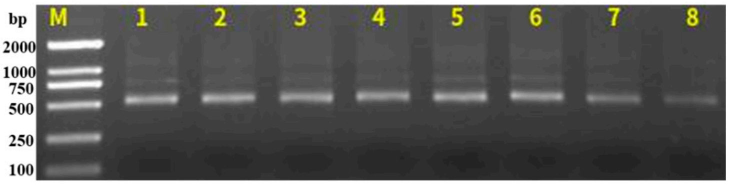

Figure S1. Amplification of hypervariable region of 16S rRNA gene sequence in different stages of SBR reactor
